# Improving Treatment Through Research: Directing Attention to the Role of Development in Adolescent Treatment Success

**Published:** 2009

**Authors:** Eric F. Wagner

**Keywords:** Underage drinking, adolescent, treatment method, treatment outcome, psychological development, clinical trials, literature review

## Abstract

Treatment of adolescents with alcohol use problems can be as successful as in adults, but the success often is short-lived, with most treated adolescents relapsing within a few months. Developmental differences among adolescents may contribute to this high rate of relapse, and treatment approaches that pay direct attention to the patients’ developmental status may improve outcomes. To date, studies assessing adolescent alcohol treatment rarely have investigated the association between developmental stage and outcome. In addition, even experts do not fully agree on the developmentally appropriate outcomes that should be evaluated in adolescent treatment studies. Research methods and variables used to assess the outcome of adolescent alcohol treatment often rely on those used in treatment studies of adults. Developmental factors that may directly influence adolescents’ amenability to treatment, such as pubertal status, psychological development (e.g., executive mental functions), social relationships, and developmental transitions, have not been adequately investigated. Studies using concepts from developmental science are needed to determine how individual characteristics, various contextual influences (e.g., from peers, family, or the social environment), and the interactions of these factors influence alcohol use behavior, amenability to treatment, and treatment outcomes. Knowledge gained in studies directly examining developmental factors should help in the design of more effective treatment programs with lower relapse rates.

As the preceding articles in this journal issue have shown, alcohol use and abuse among adolescents is a significant public health problem. Accordingly, many adolescents require treatment for drinking problems. Existing studies have indicated that treatment of teenagers with alcohol and other drug (AOD) use problems can be as successful as treatment of adults with similar problems and can improve the adolescents’ functioning in a variety of domains (e.g., school performance, ability to cope with emotional distress, and family relationships). Moreover, different treatment approaches do not appear to differ from each other in their likelihood to produce successful outcomes (Brown et al. 1996; Catalano et al. 1990; Faden 2006; Wagner et al. 1999; Williams and Chang 2000).

Additional studies, however, have indicated that adolescent treatment successes may be short-lived because about half of all teenagers treated for AOD use problems relapse within 3 months of treatment completion and two-thirds relapse within 6 months (Brown et al. 1989, 1990; Latimer et al. 2000). To reduce these relapse rates and the resulting burden on the individual patient, his or her family, and society, it is important to determine the reasons underlying this limited treatment success and to develop strategies to improve long-term treatment outcomes. One reason for this limited treatment success may be that treatment programs do not direct enough attention to developmental issues that potentially influence response to treatment. Adolescence is a period of enormous emotional, psychological, physiological, and social changes; accordingly, the treatment needs and responses of an adolescent at an earlier developmental stage can differ vastly from those of a teenager at a later developmental stage. Yet researchers currently know almost nothing about how developmental issues may influence treatment responses among adolescents with AOD problems.

A better understanding of the possible associations between developmental stage and treatment response among alcohol-abusing teenagers is vital for several reasons, including:

- The personal and environmental factors that may influence treatment response likely are similar to the ones that affect treatment outcome in adults; however, the extent of these effects and the mechanisms through which they act may depend on developmental stage (Ramo et al. 2005).
- The developmental processes and transitions that occur during adolescence (e.g., puberty, formation of a person’s identity, transition to middle school and then to high school) are distinct from the experiences of adulthood; moreover, the ways these processes and transitions influence drinking behavior differ as a teenager matures (Baer and Bray 1999; Chassin et al. 2004).
- Developmental variations and transitions can affect the pattern of an adolescent’s AOD use and, consequently, the prevalence of AOD-related problems. Simultaneously, these variations also influence the processes through which adolescents change their behaviors (e.g., the factors that can promote behavioral change) (Brown 2004).

Therefore, over the past two decades, researchers and clinicians have begun to take such developmental issues into account when treating adolescents with alcohol-related problems. It now is recognized that adolescents with alcohol problems differ distinctly from their adult counterparts and that treatment design and implementation need to take these differences into consideration to improve treatment effectiveness and reduce high relapse rates (Winters 1998). One promising strategy to achieve this goal may be to further adapt treatment approaches to the different developmental stages of patients (i.e., increase the developmental sensitivity of the programs). Despite some progress in this area, however, there still persists a shortage of effective, evidence-based interventions to treat AOD use disorders among adolescents (Cornelius 2005).

Another suggested strategy has been to promote community- and school-based interventions, which might have two beneficial effects. First, these approaches may be more developmentally appropriate for adolescents than traditional clinic-based treatments (Brown et al. 2005; Wagner et al. 2000, 2004). Second, they also may be able to address a second major problem in the treatment of adolescents with AOD problems—namely, that the vast majority of adolescents with such problems receive no treatment at all. In fact, some studies have estimated that only 10 percent of adolescents with AOD use problems receive any treatment (Clark et al. 2002; Dennis et al. 2003) and adolescents from ethnic minorities or economically disadvantaged backgrounds may be especially underserved (Aguirre-Molina and Caetano 1994; Giachello 1994; Neighbors 1985). These adolescents in particular may be easier to reach with school- or community-based interventions than with clinic-based programs.

For these reasons, interest in developmental issues that may affect treatment effectiveness among adolescents and in the design of developmentally appropriate treatment approaches has increased considerably in recent years. This article reviews the extent to which developmental stage has been considered in the existing literature on treatment of adolescents with AOD use problems. It also introduces some of the developmental issues and processes that most likely affect the outcome of adolescents receiving treatment for AOD use problems and discusses how concepts and methods from applied developmental science can be integrated directly into research on adolescent AOD treatment outcome.

## Age and Grade as Proxies for Developmental Level

To determine the extent to which developmental issues have been considered in studies on the effectiveness of treatment of adolescents, a literature search was conducted of studies published since 1990 that reported findings of clinical trials with adolescents with alcohol use problems (for more information, see Wagner 2008). Each of the selected studies was evaluated with respect to the following:

- What age-groups were included in the study? The National Institute on Alcohol Abuse and Alcoholism (NIAAA) Underage Drinking Research Initiative classifies adolescents in three age-groups—less than 10 years of age, 10–15 years of age, and 16–20 years of age—to organize the knowledge base on alcohol use and its consequences among adolescents; these three categories also were used to analyze the clinical trials identified in this literature review.
- What other information on the developmental level of the study participants was provided that could be related to treatment effects?

The literature review found that even in the best of cases, the only information provided on the developmental level was the mean age of the study participants, the standard deviation (SD) from the mean, and the age range. Moreover, virtually no studies directly examined how age affects treatment response.

### Age Range of Adolescents in the Studies

Overall, the review of the literature found that older teenagers (i.e., ages 16–20) predominated in the studies. For example, in a study by Kelly and colleagues (2000), approximately 84 percent of the participants were ages 15 or older; in a study by Tait and colleagues (2004), 86 percent of participants were ages 15 or older; and in a study by Winters and colleagues (2000), 60 percent were ages 16 and older.

Overall, the review found that only approximately 15 percent of all adolescents studied were younger than 15 years of age, and none were younger than 12 years of age. Thus, the findings of most published studies of adolescent alcohol abuse treatment are mainly derived from the oldest age-group of adolescents. And although it is not surprising that children from the youngest age-group (i.e., less than 10 years of age) are not included in treatment studies because it is unlikely that children of this age-group have had enough exposure to alcohol to develop alcohol-related problems, the scarcity of adolescents ages 10–15 is more worrisome. Children in this age-group, which includes the middle-school years, typically begin to show differences in alcohol use patterns that are related to their risk of developing drinking problems. For example, initiation of alcohol use during the transition from elementary school to middle school, current drinking in middle school, and heavy episodic drinking in high school all have been associated with the development of drinking problems later in adolescence (Guo et al. 2000).

### Analysis of Relationship Between Age and Treatment Response

Of all the studies analyzed regarding the treatment of adolescents for alcohol use problems, only three directly examined how age affected treatment response, with the following results:

- Kelly and colleagues (2000) reported that no relationship existed between age and AOD use outcomes.
- Winters and colleagues (2000) found no significant differences between younger (ages 12–15) and older (ages 16–18) adolescents with respect to drug use frequency after treatment.
- Blood and Cornwall (1994) detected no differences in age between adolescents who completed treatment and those who did not.

Thus, from these findings it appears that age is not related to treatment outcome. However, the validity of this interpretation is limited by the small number of studies, predominance of older adolescents in the three study samples, and low statistical power of the study analyses (i.e., relatively small number of adolescents participating in each study and insensitive data analyses).

Another concern regarding the generalizability of these findings is that age alone may not be a reliable indicator of developmental stage—for example, some 15-year-olds may be much more advanced in their physical, social, and emotional development than others. As an alternative, some studies have used the participants’ grade levels as a marker for developmental stage in addition to analyzing age data. For example, Brown and colleagues (2005) reported both the grade and age distributions of the participants in a study assessing a school-based intervention among 9th to 12th graders. The investigators found that both age and grade were related to lifetime alcohol consumption at baseline (i.e., older teenagers and those in higher grades reported greater lifetime consumption than younger participants); moreover, grade level was more strongly related to alcohol involvement than age, suggesting that grade may be a better indicator of developmental stage than age. However, the study did not analyze the relationship between age or grade and treatment outcome.

In summary, the existing studies of treatment success in adolescents suffer from two main drawbacks. First, they primarily focus on older adolescents (i.e., ages 16 and older) and do not adequately represent younger adolescents (i.e., ages 10–15). Second, they do not directly examine the relationship between age/developmental stage and treatment response. These issues need to be addressed in future studies to gain more meaningful results.

## Developmentally Appropriate Outcomes

Another issue requiring further investigation is what kinds of outcomes should be evaluated in studies of AOD treatment for adolescents and how these outcomes can be assessed in a developmentally appropriate manner. For example, one goal of interventions among adolescents should be to interfere with the factors and processes that may predict future alcohol use problems, such as their susceptibility to peer pressure to misbehave, exposure to peer alcohol use and abuse, and personal alcohol use, all of which increase steadily throughout adolescence and mutually influence each other (Schulenberg and Maggs 2001). In other words, adolescents’ developmental levels influence their patterns of risk factors and alcohol use, as well as the interactions between risk factors and alcohol use. For example, older adolescents are more likely than younger adolescents to spend time outside the home with peers and to be invited to parties where alcohol is available and may therefore be more likely to engage in binge drinking. At the same time, older adolescents typically can drive, and the increased likelihood of drinking combined with the ability to drive can increase the risk of alcohol-related negative consequences, such as motor vehicle crashes or other alcohol-related legal problems. Therefore, a goal of treatment would be to interfere with this web of influences, thereby reducing risk of future alcohol problems.

To achieve this goal, it is essential to use sound measures of predictors of alcohol use, processes contributing to alcohol use, and alcohol-related outcomes; moreover, the assessment procedures used to determine these measures need to be developmentally appropriate.

To date, few assessment strategies have been specifically developed for adolescents. However, some investigators (Brown 2004) have recommended that adolescents should be questioned using a more informal and nonacademic style than adults, arguing that adolescents receiving school-based treatment for alcohol problems also are likely to have academic problems and deficits in interpersonal skills that could interfere with their ability to respond correctly to a “traditional” style of questioning. In addition, because self-reports can be unreliable in this age-group, it may be helpful to use assessment tools that solicit information on drinking behaviors through different formats or to use several tools with different formats to obtain accurate information (Brown 2004).

Other investigators (Hays and Ellickson 1996) attempted to identify developmentally appropriate outcomes for adolescents with alcohol problems by convening a panel of 10 experts on adolescent and adult alcohol use. These experts, however, disagreed considerably on what indicators of alcohol misuse they considered appropriate outcomes for assessing treatment effectiveness in adolescents. The experts looked at three groups of outcomes:

- Quantity–frequency criteria (e.g., frequency of drinking in the past year or in the past 30 days);
- High-risk drinking criteria (such as binge drinking, getting drunk in public places, using alcohol with other medications or drugs, or driving after drinking); and
- Negative-consequences criteria (such as missing school or work, feeling sick or having trouble concentrating, being arrested, or having an accident after drinking).

Very little agreement was found among the experts regarding the usefulness of quantity–frequency criteria, even though these measures typically are the primary outcomes reported in treatment studies among adolescents (Hays and Ellickson 1996). The experts did agree, however, that the cutoff values for distinguishing alcohol use from alcohol misuse should vary according to age, with higher cutoff values for older adolescents. Moreover, the experts agreed that indicators representing high-risk drinking and negative consequences of drinking were appropriate outcomes for assessing treatment effectiveness.

These high-risk drinking and negative-consequences criteria, however, are not used in studies of adolescent alcoholism treatment as often as quantity–frequency criteria. Outcomes that have been reported include the following:

- Categories related to the quantity–frequency measures (e.g., abstainer, minor lapser, relapse);
- Categories that combine quantity–frequency information and diagnostic criteria, such as those specified in the *Diagnostic and Statistical Manual of Mental Disorders, Fourth Edition* (DSM–IV) (e.g., abstainer, current drinker, alcohol abuser, and alcohol dependent);
- Harm reduction outcomes (e.g., reduction in number of drinks consumed per drinking occasion);
- Specific high-risk drinking behaviors (e.g., binge drinking);
- Scales measuring negative consequences of drinking (e.g., trouble at school or problems with the family);
- Psychological correlates of alcohol use problems (e.g., craving for alcohol); and
- Health risk behaviors associated with alcohol use (e.g., motor vehicle accidents, other drug use, or unprotected sex).

However, it is unknown whether any of these measures really represent developmentally appropriate consequences of adolescent alcohol use because most of them are derived from outcomes measured in alcoholism treatment of adults. Only instruments that measure negative consequences typically assess developmentally relevant events or issues (e.g., alcohol-related problems at school).

Conversely, the usefulness of symptoms such as those specified in the diagnostic criteria of the DSM–IV is the subject of much debate. For example, some of those criteria (e.g., presence of alcohol-related medical problems) are unlikely to occur in adolescents who typically have short drinking histories. Other symptoms (e.g., alcohol-related legal problems) appear to occur mainly in particular subgroups of adolescents (e.g., older male adolescents with conduct disorder) (Martin and Winters 1998).

Because even experts agree so little on what constitutes developmentally appropriate measures of adolescent alcohol use and misuse, a range of other indicators of treatment response has been reported in various studies. The use of different indicators of treatment outcome by different investigators, however, makes it very difficult to compare treatment effects among studies, particularly because these indicators often reflect fundamental differences in how adolescent treatment outcome is measured. Thus, it is of the utmost importance to specify more clearly and consistently what constitutes developmentally appropriate measures of adolescent alcohol use and treatment outcome.

In summary, for many people AOD use begins during early adolescence, increases during late adolescence, and declines during adulthood (Chassin et al. 2004), proving that developmental level clearly has an influence on AOD use patterns, particularly in adolescents. Nevertheless, few developmentally sensitive measurement strategies for assessing adolescent AOD use exist, and no agreement exists on what constitutes appropriate measures of AOD problems and treatment outcomes. Particularly for those measures that are most commonly used in treatment studies—that is, quantity–frequency measures—there is little agreement regarding their appropriateness. Thus, estimating adolescent drinking problems remains a rather imprecise process that urgently requires development of more appropriate measures and methods.

## DIRECTing Attention to Development: Suggested Variables and Methods

The acronym DIRECT is shorthand for “Developmentally Informed Research on the Effectiveness of Clinical Trials.” The overarching thesis of this review is that taking a DIRECT approach to assessing developmental level and variables in longitudinal clinical trials ultimately will lead to improved treatment outcomes for adolescents with alcohol problems. Inherent in the DIRECT approach is the assumption that developmental level likely affects amenability to treatment; one reason why many adolescents are not amenable to treatment and/or relapse within a few months of treatment completion may be related to the enormous developmental diversity among adolescents with alcohol use problems. Knowing the developmental factors that influence amenability to treatment should help researchers design treatment programs or program components with improved chances of success.

Differences in amenability to treatment among adolescents rarely have been analyzed in the treatment literature. As previously described, to date only three studies (Winters et al. 2000; Kelly et al. 2000; Blood and Cornwall 1994) have evaluated the association between developmental stage and treatment outcome, and even those studies were limited to analyses of the association between age (which is an imperfect indicator of developmental level) and drinking outcomes and found no association between the two. Moreover, although a large body of research exists on adolescent development and developmental psychopathology, this knowledge has only rarely been incorporated into adolescent alcohol abuse treatment research (Cicchetti and Rogosch 2002; Lerner and Steinberg 2004; Steinberg 2002). As a result, researchers do not know whether or when development–treatment interactions occur in adolescents treated for alcohol problems.

To allow for more DIRECT analyses of how developmental issues may influence adolescents’ responses to alcohol treatment, the following list has been developed of developmental processes and transitions that may influence adolescent behavior, including alcohol use (Wagner 2008). These influences fall into four categories:

- Biological factors, such as pubertal status and timing (e.g., if and when menarche has occurred), hormonal changes, physical appearance and size, and maturation of brain regions involved in decisionmaking and other relevant cognitive functions (i.e., prefrontal cortex and limbic system);
- Psychological factors, such as identity formation, problem-solving skills, self-regulation, executive mental functions (e.g., planning, decision-making, reasoning skills), cognitive capacity, or moral reasoning;
- Social factors, such as peer, sibling, and parental influences; intimacy and sexual involvement; interpersonal negotiation and social problem solving; or use of media and information sources; and
- Transitions, such as moving from elementary to middle school or from middle school to high school, getting a driver’s license, starting a job, or losing one’s virginity.

Most of these factors have been shown to be modestly associated with adolescent alcohol use; nevertheless, none of them have been examined as potential influences on adolescents’ responses to treatment for alcohol problems. The following paragraphs describe some of these developmental constructs in more detail.

### Roles of Developmental Influences in Shaping Adolescent AOD Use and Treatment Response

#### Puberty

Susman and Rogol (2004) found that AOD use is more prevalent in boys and girls who enter puberty early than in those who enter it at the “normal” age or later. Moreover, in adolescent girls, early menarche was associated with earlier drinking initiation and more frequent alcohol use (Dick et al. 2000). Overall, however, the influence of pubertal status or timing on AOD use appears to be small, and many other factors related to this highly complex developmental period appear to moderate the relationships among pubertal status, psychological development, behavior, and their interactions (Susman and Rogol 2004).

#### Maturation of the Brain

During adolescence, a region at the front of the brain (i.e., the prefrontal cortex) continues to develop, enabling it to integrate cognitive functions and regulate emotions, attention, and behavior. This leads to the development of a more fully conscious, self-directed, and self-regulating mind (Keating 2004). Individual differences in these developmental processes and their outcomes may affect the development of psychopathology, including, presumably, AOD use problems (Keating 2004). Consistent with this hypothesis, imaging studies (Brown et al. 2000; Moss et al. 1994; Schweinsburg et al. 2005; Tapert et al. 2002, 2004) found that adolescents who drink heavily show deficits in certain cognitive and brain responses, especially if they also use marijuana. It is not yet known, however, if these deficits precede AOD involvement or if they are a consequence of AOD use. The role such deficits may play in adolescents’ treatment responses also still needs to be clarified.

#### Changes in Social Functioning

One of the hallmarks of adolescence that also may impact alcohol use behavior and the response to treatment for alcohol problems is the changing relationship the adolescent has with his or her peers and parents. During adolescence, peer influences on adolescent behavior increase, and these relationships grow increasingly complex. These changes also impact adolescents’ AOD use behavior, and studies consistently find that AOD use by peers is strongly associated with an adolescent’s AOD use (Chassin et al. 2004). Moreover, peer AOD use predicts relapse in adolescents who have been treated for AOD use. In fact, 90 percent of relapses among adolescents occur when they are together with other people and are exposed to direct or indirect social pressure to use AODs (Brown et al. 1989). The exact mechanisms through which peer use affects adolescent AOD use are not clear, however. For example, it is possible that adolescents who are predisposed to using alcohol or drugs may join peer groups who already are using AODs. Alternatively, peer influences may motivate an adolescent to try AODs even if he or she does not have a predisposition to alcohol or drug use (Chassin et al. 2004). Because of the complexity of peer relationships during adolescence, it is difficult to determine the respective contributions of various possible mechanisms.

Another characteristic development of adolescence is the change in the relationship with parents. Overall, the influence of parents recedes somewhat, as the relationship increasingly is based on negotiation and joint decision making rather than parents making most of the decisions for the child; moreover, the interdependence between parents and child declines and the parent–child relationship is considered less close (Clark et al. 2002). Nevertheless, strong associations exist between adolescent AOD use and the familial environment, including parenting style, family climate, the parent–adolescent relationship, and the standards and attitudes that parents convey regarding AOD use (Chassin et al. 2004). The role that parent–child relationships and their changes during adolescence play in treatment responses, however, has not yet been elucidated.

#### Developmental Transitions

Certain transitions are unique to adolescence, such as the entry into middle school or high school, which are accompanied by complex changes in the adolescents’ social and academic environment. These transitions likely also have some impact on AOD use behavior, given that they coincide with the time period during which adolescents typically have their first experiences with alcohol or other drugs (i.e., between grades 7 and 10) (Chassin et al. 2004). To date, however, no studies have examined how these transitions may influence treatment outcome, even though most adolescents in treatment for AOD use problems are 16 years old—the age at which most students enter high school.

Another transition characteristic of adolescence is the beginning of working outside the home. Studies found that the more hours adolescents work outside the house, the more likely they are to use alcohol and cigarettes, particularly if work hours exceed those spent on school-related and other activities (Staff et al. 2004). Again, however, no studies have assessed the influence of the transition to work on treatment and treatment outcome.

### How Can the DIRECT Approach Be Incorporated Into Adolescent Treatment Outcome Research?

When attempting to study adolescents’ response to treatment for AOD use problems, it is important to realize that the developmental influences described above do not act in isolation to influence adolescent AOD use (and possibly treatment response) but often are interrelated. For example, hormonal changes and pubertal development may lead to the characteristic mood swings of adolescence (i.e., negative affect), which may adversely affect parent–child relationships, thereby contributing to AOD use and ultimately decreasing response to treatment (Brown 1993). This complex web of influences and their interactions has not yet been untangled, and researchers have yet to determine which variables are particularly important in shaping AOD use and the outcome of treatment for AOD use problems.

Because of the complexity of these interrelationships, however, it is difficult to conduct developmentally informed research that takes all of these variables into consideration and applies concepts and methods of developmental psychology and psychopathology to adolescent alcoholism treatment research (for more information, see Cicchetti and Rogosch 2002; Lerner and Steinberg 2004). One model that can inform and guide treatment research among adolescents is known as developmental systems orientation (Lerner 2002). This model involves three key assumptions:

- Adolescents (and other individuals) are embedded in multiple interrelated contexts (e.g., family, school, peers, community, and culture) that all shape adolescents’ experiences and behaviors.
- The relationship between the adolescent and these contexts is bidirectional—that is, the adolescent is not only influenced by, but also influences, the people and events that are part of these contexts.
- Adolescents actively contribute to their development through their personal characteristics, desires, and needs that determine how the adolescents interact with the environment.

Thus, from the perspective of this model, adolescent development (including AOD use behaviors and response to AOD treatment) is determined by the numerous changing relationships between the adolescent and the changing and interdependent contexts in which he or she is embedded.

Schulenberg (2006) reviewed research approaches that seek to understand adolescent development and the evolution of risk behaviors, such as alcohol use, and to guide treatment research for adolescents with AOD use problems. He noted the following:

- The adolescent is embedded into multiple contexts that can act in a hierarchical manner. For example, the adolescent is directly influenced by primary contexts (e.g., the family or peer group), which in turn are exposed to and influenced by larger-scale contexts (e.g., social, technological, political, or economic systems); moreover, all of these multiple contexts interact with each other.
- In shaping the adolescent’s behavior, the various contexts also interact with individual characteristics (e.g., the ability to regulate his or her behavior), and as the adolescent matures, these individual characteristics, contexts, and their interactions may demonstrate both continuities and discontinuities.
- Contexts and individuals interact in a dynamic manner; as a result, factors that predict risk behavior (e.g., the proportion of an adolescent’s peers that use alcohol) and outcomes (e.g., the adolescent’s own alcohol use) influence one another reciprocally in an ongoing interplay of variables. Thus, the cause-and-effect relationship between risk factors and outcomes may actually reverse over time—for example, although alcohol use by peers may initially contribute to alcohol use by the adolescent, the adolescent’s drinking behavior may eventually lead him or her to seek out specifically those peers who condone AOD use.
- A single developmental context factor can lead to a variety of outcomes; conversely, different context factors also can lead to the same outcome.
- Risk behaviors tend to build on each other; for example, early risk behaviors (e.g., legal cigarette smoking) tend to influence subsequent risk behaviors (e.g., illegal AOD use).

Mathematical modeling approaches are available that can account for this complexity and variability in developmental processes. For example, an approach called growth mixture modeling, in which data are collected on at least three different occasions (e.g., before treatment, after treatment, and after one or more follow-up periods), allows researchers to consider how multiple behaviors interact over time (Schulenberg 2006). Although such longitudinal research approaches also have limitations, growth mixture modeling appears to be the best available approach for understanding how changes in context are related to changing person–context interactions and how they influence individual risk behavior and outcomes.

Many alcoholism treatment researchers focusing on adolescents, however, are unaccustomed to applying these concepts of applied developmental science. Accordingly, typical clinical trials of treatments for AOD use problems, while collecting data at the time points indicated above, do not routinely examine how adolescents’ individual characteristics, contexts, and alcohol-related outcomes interact and change over time. Therefore, efforts to incorporate applied developmental science methods into studies of treatment effectiveness for adolescents with AOD use problems are urgently needed so that researchers and clinicians can better understand how developmental issues may influence the course of treatment and treatment outcomes of these adolescents.

## Existing Developmental Models of Adolescent Addiction

Another source of knowledge that may potentially inform the development and assessment of alcoholism treatment for adolescents and the evaluation of its effectiveness are models of how addiction to AODs develops in adolescents in the first place. Several developmental models of the causes of adolescent alcohol use and its associated problems exist. Some of these models have emphasized the contribution of individual factors or influences, such as deficits in coping with developmental tasks (Schulenberg et al. 1996), risk perception (Hampson et al. 2001), cognitive and emotional states (Brown 2001; Metrik et al. 2004), or social influences (e.g., by parents, siblings, or peers) (Windle 2000). Three other types of models focus on the role of developmental transitions and challenges in the development of psychopathological disorders, which may include AOD use problems (Graber 2004). These models posit the following:

- People are more likely to demonstrate negative behavior if they experience two or more major life events or transitions at the same time or shortly after each other;
- Developmental transitions exacerbate problems that already exist (e.g., alcohol use); or
- People are more sensitive to environmental or contextual influences when they are experiencing rapid changes, such as those occurring during adolescence.

Some of these models also propose that some people may have characteristics predisposing them to heightened sensitivity to such developmental transitions.

To date, however, researchers have not incorporated any of these models of risk for adolescent addiction in studies of the effectiveness of treatment for adolescent alcohol use problems; accordingly, their relevance and the relative contributions of these various factors to adolescent treatment outcome remain unknown.

## Conclusions

Although treatment of adolescents for alcohol use problems can be effective, most treated adolescents relapse to drinking and other drug use within 6 months of treatment completion. This dismal record might be improved if researchers took the developmental status of the affected adolescents into consideration when designing treatment interventions for this age-group. Ample research has demonstrated that numerous developmental variables can influence alcohol-related characteristics, such as pattern of use, prevalence of alcohol-related problems, patterns of risk for alcohol-related problems, and the pathways through which adolescents can change their behaviors and maintain those changes. Unfortunately, the influence of these developmental variables has yet to be fully assessed in studies examining treatment effectiveness for adolescents with alcohol use problems.

To improve treatment of adolescents with alcohol use problems as well as studies assessing the effectiveness of such treatments, clinical researchers should begin to incorporate the DIRECT approach in studies with teenagers, rather than rely so heavily on models and methods borrowed from research on adults with alcohol problems. For various reasons—for example, lack of well-specified developmentally sensitive measurement strategies and behavioral assessments, lack of knowledge about these perspectives among researchers, and the complexity of developmental research—such studies rarely have been undertaken. Only through clinical trials of adolescent alcohol treatment that are longitudinal, include information on developmental levels, and directly assess developmentally relevant variables, however, can researchers learn more about the interactions between developmental level and treatment response. Understanding the role of development in treatment success, in turn, will enable the design of approaches that benefit the full range of adolescents with alcohol problems over the long term.
